# Analysis of quality metrics in comprehensive cancer genomic profiling using a dual DNA–RNA panel

**DOI:** 10.1016/j.plabm.2024.e00368

**Published:** 2024-02-15

**Authors:** Kousuke Watanabe, Shinji Kohsaka, Kenji Tatsuno, Aya Shinozaki-Ushiku, Hideaki Isago, Hidenori Kage, Tetsuo Ushiku, Hiroyuki Aburatani, Hiroyuki Mano, Katsutoshi Oda

**Affiliations:** a Department of Clinical Laboratory, The University Tokyo, Tokyo, Japan; b Department of Respiratory Medicine, The University of Tokyo, Tokyo, Japan; c Division of Cellular Signaling, National Cancer Center Research Institute, Tokyo, Japan; d Genome Science and Medicine Laboratory, RCAST, The University of Tokyo, Tokyo, Japan; e Division of Integrative Genomics, Graduate School of Medicine, The University of Tokyo, Tokyo, Japan; f Department of Pathology, Graduate School of Medicine, The University of Tokyo, Tokyo, Japan; g Next-Generation Precision Medicine Development Laboratory, Graduate School of Medicine, The University of Tokyo, Tokyo, Japan

**Keywords:** Comprehensive cancer genomic profiling, Nucleic acid quality, Next-generation sequencing

## Abstract

**Background:**

The nucleic acid quality from formalin-fixed paraffin-embedded (FFPE) tumor vary among samples, resulting in substantial variability in the quality of comprehensive cancer genomic profiling tests. The objective of the study is to investigate how nucleic acid quality affects sequencing quality. We also examined the variations in nucleic acid quality among different hospitals or cancer types.

**Methods:**

Three nucleic acid quality metrics (ddCq, Q-value, and DV200) and five sequencing quality metrics (on-target rate, mean depth, coverage uniformity, target exon coverage, and coverage of the housekeeping gene) were examined using 585 samples from the Todai OncoPanel, a dual DNA–RNA panel.

**Results:**

In the DNA panel, ddCq served as an indicator of sequencing depth and Q-value reflected the uniformity of sequencing across different regions. It was essential to have favorable values not only for ddCq but also for Q-value to obtain ideal sequencing results. For the RNA panel, DV200 proved to be a valuable metric for assessing the coverage of the housekeeping genes. Significant inter-hospital differences were observed for DNA quality (ddCq and Q-value), but not for RNA quality (DV200). Differences were also observed among cancer types, with Q-value being the lowest in lung and the highest in cervix, while DV200 was the highest in lung and the lowest in bowel.

**Conclusions:**

We demonstrated distinct characteristics and high predictive performances of ddCq, Q-value, and DV200. Variations were observed in the nucleic acid quality across hospitals and cancer types. Further study is warranted on preanalytical factors in comprehensive cancer genomic profiling tests.

## List of abbreviations

FFPE: formalin-fixed paraffin-embedded
Cq: quantification cycle
ddCq: delta-delta Cq
DV200: distribution value 200
NGS: next-generation sequencing

## 1 Introduction

Comprehensive cancer genomic profiling (CGP) tests have been increasing used in clinical practice to guide treatment for patients with cancer. Quality control of CGP tests is a complex process due to the numerous steps involved, including sample collection, nucleic acid extraction, library preparation, sequencing, and bioinformatics process. As the quality of nucleic acids from formalin-fixed paraffin-embedded (FFPE) tumor varies widely among samples and directly affects the quality of downstream analyses [1], it is necessary to monitor the quality metrics at each step to access the success or failure of CGP tests.

Different metrics are used to measure DNA quality, such as DNA integrity number (DIN), Q-value, and ddCq. DIN is a numerical measurement ranging from 1 to 10 determined using the Genomic DNA ScreenTape assay. The Q-value is the ratio of PCR-amplifiable DNA to double-stranded DNA developed by National Cancer Center in Japan [2]. The ddCq value is determined by real-time PCR of two amplicons of different lengths [1]. DV200, which is the proportion of RNA fragments equal to or longer than 200 nucleotides in length, is used for the quality metric of RNA. However, previous studies rarely include detailed descriptions of how these quality metrics impact downstream analysis quality, and there is no consensus on the differential use of multiple metrics for assessing DNA quality.

We have previously reported the clinical utility of Todai OncoPanel (TOP), which is a dual DNA–RNA panel that tests for 464 gene alterations with its DNA panel and 365 fusion transcripts with its RNA panel [3,4]. The purpose of this study is to investigate how nucleic acid quality affects sequencing quality metrics in the targeted sequencing using our TOP data. We also examined whether there are variations in nucleic acid quality among different hospitals or cancer types.

## 2 Materials and methods

### 2.1 Patient samples

Prospective clinical sequencing using TOP was started as a project under the Japan Agency for Medical Research and Development (AMED) in 2017 [4], and it was subsequently conducted as Advanced Medical Care B from 2018 to 2019 [3]. In the AMED project, patients were recruited from the University of Tokyo Hospital, while in Advanced Medical Care B, patients were recruited from several hospitals as previously described [3]. In the present study, the combined dataset incorporating data from the AMED project and the Advanced Medical Care B was analyzed, which included a DNA panel with 582 samples and an RNA panel with 572 samples. The fixation was carried out using 10–20% neutral buffered formalin within 24–72 h. Both projects were approved by the institutional ethics review board (protocol #G10114 and #P2017017), and informed consent was obtained from all the patients.

### 2.2 Clinical sequencing

All samples were analyzed at a single Clinical Laboratory Improvement Amendments (CLIA) compliant laboratory at the University of Tokyo according to the laboratory's standard operating procedures. Genomic DNA was isolated from FFPE samples using GeneRead DNA FFPE Kits (Qiagen). DNA quantity was determined using the Qubit Fluorometer (Thermo Fisher Scientific) for double-stranded DNA and TaqMan Copy Number Reference assay human RNase P (Thermo Fisher Scientific) for PCR-amplifiable DNA. The Q-value (the ratio of PCR-amplifiable DNA to double-stranded DNA) was calculated to evaluate the quality of DNA. DNA quality was also assessed by the ddCq value using the FFPE DNA QC Assay version 2 (Thermo Fisher Scientific). The quantity of DNA was considered as the amount of double-stranded DNA when the Q-value was equal to or greater than 1, and as the amount of PCR-amplifiable DNA when the Q-value was less than 1. Total RNA was extracted using RNeasy FFPE Kit (Qiagen) and its quality (DV200) was evaluated on a 2200 TapeStation (Agilent Technologies). The library preparation, next-generation sequencing (NGS), and data analysis were performed as previously described [3,4]. In brief, the libraries of the DNA and the RNA panel were prepared using a SureSelectXT Custom kit (Agilent Technologies) and a SureSelect RNA Capture kit (Agilent Technologies), respectively. NGS was carried out using the Next-seq platform (Illumina). Somatic variants, copy number alterations, and germline findings were detected with the DNA panel. Gene fusions and exon skipping were detected by the RNA panel. Details of analysis pipelines were described previously [4].

### 2.3 Quality metrics

The ddCq, Q-value, and DV200 were used as nucleic acid quality metrics. On-target rate, mean depth, coverage uniformity (percentage of target bases covered at least 0.2 times the mean depth), and target exon coverage (percentage of target bases covered by at least 200 reads) were used as the sequencing quality metrics of the DNA panel. Coverage of the housekeeping gene (percentage of housekeeping gene bases covered by at least 200 reads) was used as the sequencing quality metric of the RNA panel. To summarize, the study involved the analysis of six quality metrics for the DNA panel and two quality metrics for the RNA panel.

### 2.4 Statistical analysis

We predicted the probability distributions of each quality metrics through data observation and maximum likelihood estimation. Kolmogorov-Smirnov test was applied to confirm whether each quality metric followed a specific probability distribution. The relation between the years after sampling and nucleic acid quality metrics was analyzed using the Kruskal-Wallis test, followed by post hoc Wilcoxon rank sum tests for multiple comparisons using adjusted p-values by the Bonferroni correction method. The correlation between the nucleic acid quality metric and the sequencing quality metric was assessed using the Spearman's rank correlation coefficient. Receiver operating characteristics (ROC) analysis was preformed and the area under the curve (AUC) was calculated to validate the diagnostic performance of nucleic quality metrics. A generalized linear model was applied to identify factors contributing to each quality metrics. All data analysis was conducted in RStudio (version 1.3.1073) using the R statistical language version 4.0.2. A significance level of p < 0.01 was deemed statistically significant.

## 3 Results

### 3.1 Sample characteristics and distribution of quality metrics

A total of 585 samples (582 DNA samples and 572 RNA samples) were included in the present study. Among them, the most frequent cancer types, accounting for more than 5% of the total samples, were lung (n = 114), bowel (n = 90), ovarian/fallopian tube (n = 58), uterus (n = 51), soft tissue (n = 38), and cervix (n = 31).

First, the histograms of eight quality metrics under investigation were created to examine the probability distributions (Fig. 1 and Supplementary Fig. 1). Three parameters (ddCq, Q-value, and DV200) represent nucleic acid quality metrics, while four parameters (on-target rate, mean depth, coverage uniformity, and target exon coverage) represent sequencing quality metrics of the DNA panel. The coverage of the housekeeping gene serves as a quality metric of the RNA panel.

**Fig. 1:**
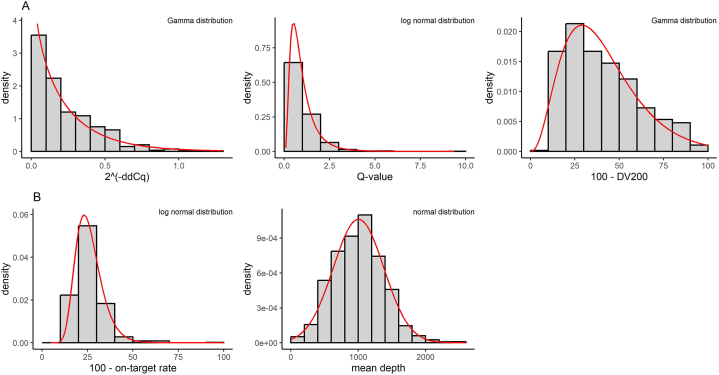
Distributions of each quality metrics. Histograms depict probability densities of nucleic acid (A) and sequencing quality metrics (B). All five metrics in this figure adhere to the probability distributions shown in the upper right corner, confirmed by Kolmogorov-Smirnov tests. Red lines indicate probability density function curves, derived from maximum likelihood estimation. (For interpretation of the references to color in this figure legend, the reader is referred to the Web version of this article.)

The mean depth followed a normal distribution (Fig. 1B), whereas the other metrics did not. According to the Kolmogorov-Smirnov test, 2^(−ddCq)^ and (100 – DV200) followed a gamma distribution, while Q-value and (100 – on-target rate) followed a log-normal distribution (Fig. 1). The remaining three metrics (coverage uniformity, target exon coverage, and coverage of the housekeeping gene) could not be fitted to a specific probability distribution (Supplementary Fig. 1).

Next, we evaluated the effect of sample storage on nucleic acid quality (Fig. 2). The nucleic acids quality metrics varied widely between samples and deteriorated significantly (p < 0.001, Kruskal-Wallis test) as the years after sampling increased. While ddCq demonstrated a gradual decline over the course of the first three years since sampling, Q-value did not exhibit significant changes over the same three-year period. DV200 showed no significant difference between the 3–5 years and the over 5 years, with 49% (26/53) of samples from over 5 years indicating values of 50 or higher. These data reaffirmed the variability in quality metrics among samples in CGP test using FFPE tumor samples.

**Fig. 2:**
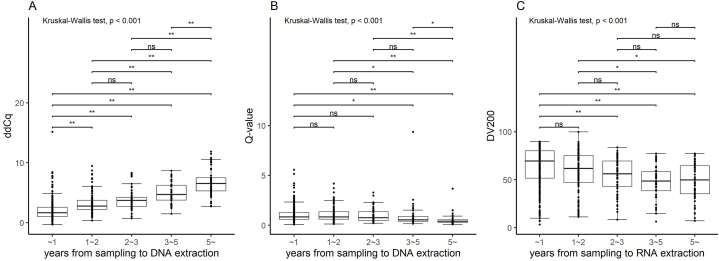
Effect of sample storage on nucleic acid quality. Each boxplot displays interquartile range (box), median (horizontal line), whiskers representing 1.5 times the interquartile range, and data points (dots) for nucleic acid quality metrics. We conducted Kruskal-Wallis tests, followed by post hoc Wilcoxon rank-sum tests for inter-group comparisons (*p < 0.01, **p < 0.001, “ns” indicates not significant).

### 3.2 Correlation between nucleic acid quality metrics and sequencing quality metrics

To investigate the correlation between nucleic acid quality metrics and sequencing quality metrics, Spearman's rank correlation coefficients were computed (Fig. 3, Fig. 4). As the quantity of the input nucleic acid used for the library preparation directly influence the sequencing quality metrics, the analysis was conducted on samples where an adequate amount of nucleic acid was obtained, and the library preparation was performed using the ideal input quantities (200 ng for DNA panel and 500 ng for RNA panel). In fact, most samples (530 samples for DNA panel and 532 samples for RNA panel) were processed using the ideal input nucleic acid quantities.

**Fig. 3:**
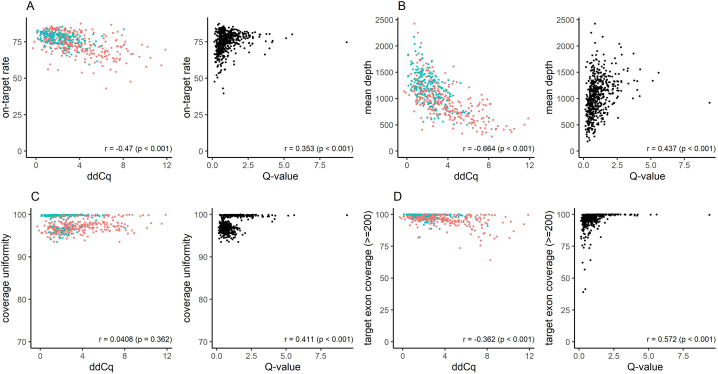
Correlation between nucleic acid quality metrics and sequencing quality metrics in the DNA panel. Scatterplots illustrate quality metrics for samples using ideal (200 ng) input DNA, with Spearman's rank correlation coefficient (r) shown in the bottom right corner. Colors indicate Q-value (blue for Q-value ≥ 1, red for Q-value < 1). (For interpretation of the references to color in this figure legend, the reader is referred to the Web version of this article.)

**Fig. 4:**
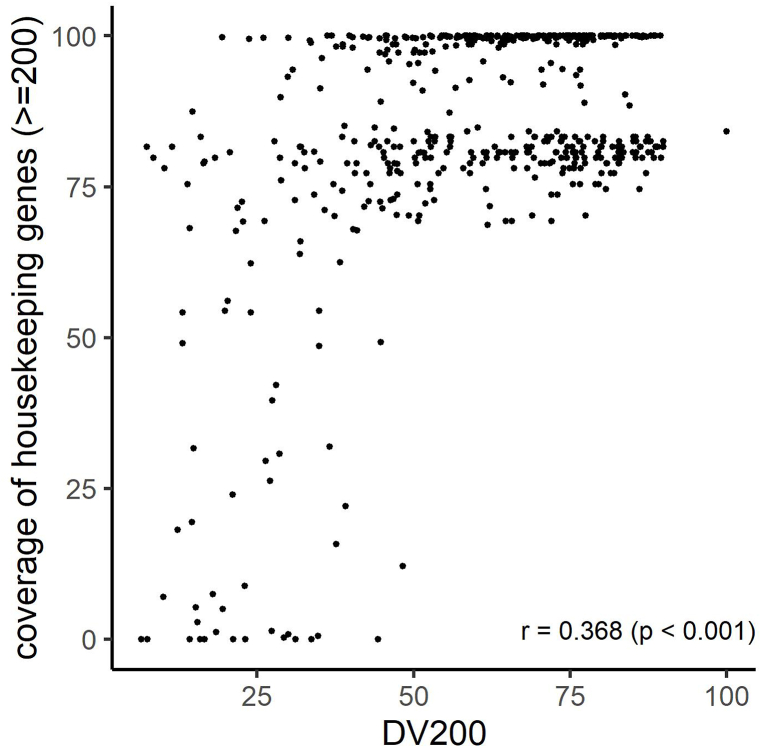
Correlation between DV200 and sequencing quality metric in the RNA panel. A scatterplot illustrates quality metrics for samples using ideal (500 ng) input RNA, with Spearman's rank correlation coefficient (r) shown in the bottom right corner.

Statistically significant correlation was observed between nucleic acid quality metrics and sequencing quality metrics in the combination excluding ddCq and coverage uniformity. The coverage uniformity was correlated with Q-value, but unrelated to ddCq, suggesting that ddCq and Q-value possess distinct characteristics (Fig. 3C). For the RNA panel, a significant correlation was observed between DV200 and the coverage of the housekeeping gene (Fig. 4).

### 3.3 Generalized linear model for sequencing quality metrics

A multivariate analysis was conducted to further investigate the differences between ddCq and Q-value (Supplementary Table 1A). As the two sequencing quality metrics, on-target rate and mean depth, exhibited specific probability distributions (Fig. 1B), a generalized linear model was applied using three explanatory variables: Q-value, ddCq, and the time after sampling. On-target rate showed a significant association only with ddCq (Supplementary Table 1A and Supplementary Fig. 2), while mean depth showed significant associations with both Q-value and ddCq (Supplementary Table 1A). Regarding mean depth, an increase of 1 in ddCq was associated with an increase of 42, while an increase of 1 in ddCq resulted in a decrease of 105. Neither on-target rate nor mean depth had any relationship with the time after sampling. These data demonstrate that if both DNA quality metrics are favorable, satisfactory sequencing results can be obtained regardless of the time after sampling.

### 3.4 Predictive performance of ddCq, Q-value, and DV200

**Fig. 5:**
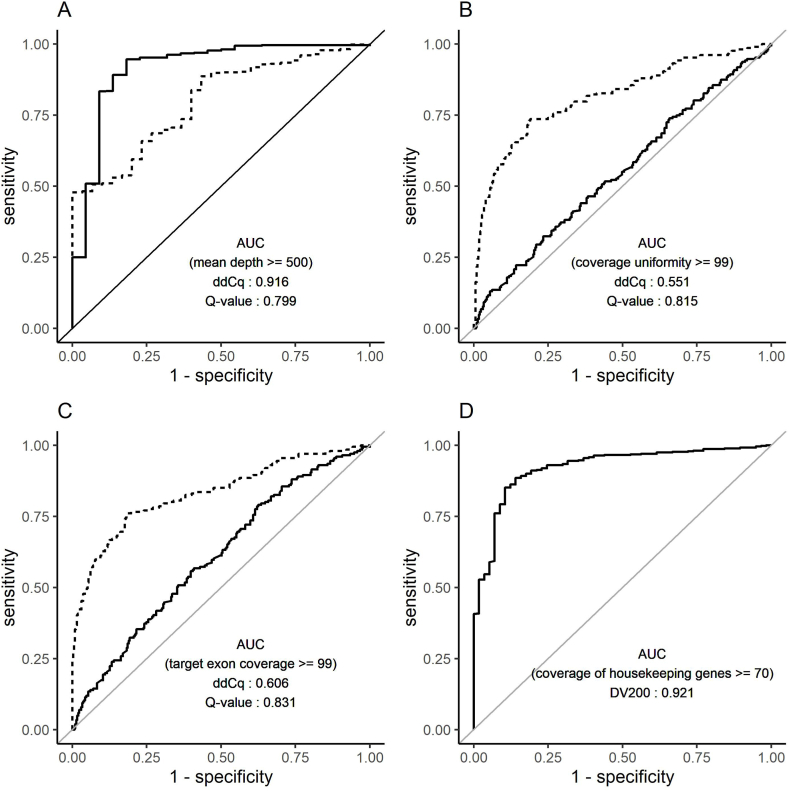
Predictive performance of ddCq, Q-value, and DV200. ROC analysis of mean depth (A), coverage uniformity (B), target exon coverage (C), and coverage of the housekeeping genes (D). The solid and dashed lines in Fig. 5A–C represent ROC curves for ddCq and Q values, respectively.

ROC analysis was conducted to investigate the predictive performance of ddCq, Q-value, and DV200 (Fig. 5). The AUC values were calculated to quantify the discriminatory power of the variables in distinguishing whether the mean depth is greater than or equal to 500, whether the coverage uniformity is greater than or equal to 99%, whether target exon coverage is greater than or equal to 99%, and whether the coverage of the housekeeping genes is higher than 70%. In predicting mean depth, the optimal threshold for ddCq was found to be 5.36 with an AUC of 0.916 (Fig. 5A). For predicting coverage uniformity or target exon coverage, the optimal threshold for Q-value was determined to be 0.928 with AUCs of 0.815 and 0.831, respectively (Fig. 5B–C). Additionally, in predicting coverage of the housekeeping genes, the optimal threshold for DV200 was identified as 41 with an AUC of 0.921 (Fig. 5D). In the DNA panel, the proportions of ddCq ≤ 5.36 and Q-value ≥ 0.928 were 84.4% and 38.6%, respectively. For the RNA panel, the proportions of DV200 ≥ 41 was 80.1%.

To visualize the differences between Q-value and ddCq, scatter plots were created (Supplementary Fig. 3). We observed samples with favorable (low) ddCq values but unfavorable (low) Q-values (Supplementary Fig. 3A). In these cases, the on-target rate and mean depth were generally satisfactory (Supplementary Figs. 3B–3C). However, the uniformity was poor (Supplementary Fig. 3D), resulting in a decreased target exon coverage (Supplementary Fig. 3E). As demonstrated in Supplementary Fig. 3A, Q-values were frequently below the cutoff threshold even for new specimens within one year of sampling. If limited to samples within one year of sampling (DNA: 310 samples, RNA: 302 samples), the proportions of ddCq ≤ 5.36, Q-value ≥ 0.928, and DV200 ≥ 41 were 95.6%, 43.0% and 85.3%, respectively.

In summary, ddCq served as a reliable indicator of the extent of sequencing depth, Q-value reflected the uniformity of sequencing across different regions, and DV200 proved to be a valuable metric for assessing the coverage of the housekeeping genes. In the DNA panel, it was essential to have favorable values not only for ddCq but also for Q-value to obtain ideal sequencing results.

### 3.5 Variation in nucleic acid quality across hospitals and cancer types

**Fig. 6:**
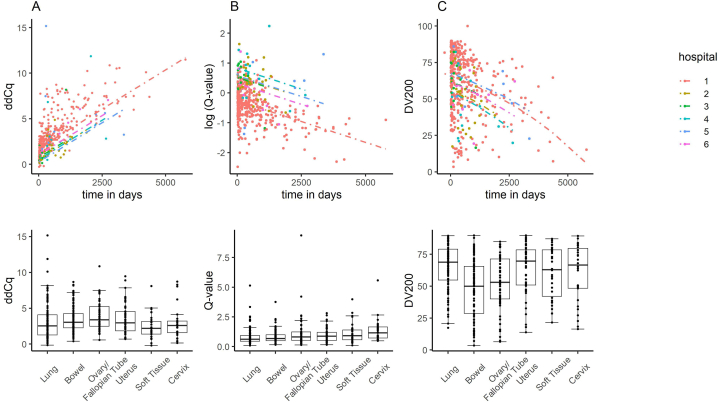
Nucleic acid quality among hospital and cancer types. The upper scatterplots depict the time since sampling and quality metrics color-coded by hospitals, along with generalized linear model regression lines. Below, boxplots represent quality metrics categorized by cancer types, including the interquartile range (box), median (horizontal line), and whiskers (1.5 times the interquartile range), and data points (dots). (For interpretation of the references to color in this figure legend, the reader is referred to the Web version of this article.)

The differences in nucleic acid quality metrics based on the sampling hospitals was investigated using multivariate analysis. Six hospitals that had submitted more than ten samples were selected for the analysis, comprising a total of 492 DNA samples and 486 RNA samples. A generalized linear model was constructed to explain the nucleic acid quality metrics using the two explanatory variables: the time after sampling and sampling hospital (Supplementary Table 1B). As shown in Fig. 6A–B, significant inter-hospital differences in ddCq and Q-value were observed. As for DV200, no significant difference was found between hospitals, but a trend of lower DV200 values was observed in hospital 2 (p = 0.024) (Fig. 6C).

Finally, we examined variations in nucleic acid quality metrics based on cancer types. We focused on the six most prevalent cancer types (lung, bowel, ovarian/fallopian tube, uterus, soft tissue, and cervix) (boxplots in the lower part of Fig. 6). A generalized linear model was constructed to explain the nucleic acid quality metrics using the three explanatory variables: the time after sampling, sampling hospital, and cancer type (Supplementary Table 1B). For the generalize linear model, the samples of the six most prevalent cancer types collected at the six aforementioned hospitals were used for the analysis (DNA: 329 samples, RNA: 326 samples). Even after introducing the explanatory variable of cancer type, significant inter-hospital differences remain evident in both ddCq and Q values. Furthermore, significant differences based on cancer type were identified in Q values and DV200. As depicted in Fig. 6B–C and Supplementary Table 1B, the Q value was lowest in lung cancer, while ovarian/fallopian, uterus, and cervix cancers exhibited significantly higher values. On the other hand, DV200 was highest in lung cancer, but demonstrated significantly lower values in bowel and ovarian/fallopian cancers. Based on the above findings, both the sampling hospital and cancer type were identified as factors associated with the variability of the nucleic acid quality.

## 4 Discussion

The quality of nucleic acid from FFPE samples vary considerably among samples, resulting in substantial inter-sample variability in the quality of CGP tests. Quality metrics of nucleic acids and sequencing data are used for quality control of CGP tests; however, detailed analysis regarding the specific relationship between these metrics remain largely unreported in the literature. Here, we revealed distinct characteristics and predictive performances of three nucleic acid quality metrics: ddCq and Q-value, and DV200. We also observed variation in the nucleic acid quality across hospitals and cancer types.

The three most common techniques for DNA quantification are UV spectrometry, fluorescent dye-based double-stranded DNA quantification, and quantitative PCR. The DNA concentration exhibits significant variability across the three methods [5], and the Q-value is the ratio of PCR-amplifiable DNA to double-stranded DNA. In the present study, samples with low Q-values exhibited low uniformity, resulting in a decreased target exon coverage, even if ddCq values were favorable (low). This result is consistent with the recent report that the amount of PCR-amplifiable input DNA predicted library complexity better than the input measured using fluorescent dye-based method [6]. Both ddCq and Q-value are valuable for predicting sequencing quality, and their combined use may aid in the interpretation of CGP results.

In this study, the optimal thresholds for ddCq, Q-value, and DV200 were determined through ROC analysis as 5.36, 0.928, and 41 respectively. For ddCq and DV200, over 80% of the samples met the cutoff criteria, confirming the feasibility of RNA analysis in FFPE samples. However, Q-value met the cutoff criteria in only 43.0% of the specimens even within a year after sampling. The cause of low Q-values in specimens within one year of sampling remains unclear. Further study is needed to investigate the factors influencing the Q-value. Additionally, the thresholds identified in the study might not necessarily represent optimal values for practical use. The inherent limitations of ROC analysis, especially the assumption of equal weights for false negatives and positives, underscore the need for cautious interpretation in real-world scenarios where the consequences of false negatives and false positives can vary.

We observed inter-hospital variations in ddCq and Q-value, suggesting that preanalytical factors at each hospital affected the DNA quality of FFPE specimens. It is reported that inter-facility variations in preanalytical factors within Southeast Asian oncology hospitals influenced nucleic acid quality [7]. Various guidelines exist on preanalytical factors for FFPE samples used in molecular testing [8], and the Japanese Society of Pathology has published a guideline for specimen handling in CGP tests [1], which covers the pre-fixation process (handling immediately after resection or collection), fixation process, and post-fixation process (decalcification, paraffin-embedding, and storage). Most of the samples used in this study were collected before 2019, prior to the regulatory approval and insurance reimbursement of CGP test in Japan. Our results suggest that inter-hospital differences may persist even at present. The limitation of this study is the lack of information on preanalytics factors, and the underlying causes of the observed inter-hospital variations remain unknown. Further prospective study incorporating various preanalytical factors would elucidate the causes of inter-hospital differences.

As far as we known, this is the first report to demonstrate differences in Q-value and DV200 based on cancer types, making it valuable information when employing a twin-panel system involving both DNA and RNA. Interestingly, in lung cancer, while RNA quality was the highest, its Q-value was the lowest. The quality metrics of DNA and RNA exhibited different distributions based on cancer types. The differences in preanalytical factors based on cancer types may have contributed to variations in nucleic acid quality.

In summary, we conducted a detailed analysis of the relationship between nucleic acid quality metrics and sequencing quality metrics and demonstrated distinct characteristics and predictive performances of ddCq, Q-value, and DV200. Furthermore, we observed variations in the nucleic acid quality across hospitals and cancer types. Further study is warranted on preanalytical factors in comprehensive cancer genomic profiling tests.

## CRediT authorship contribution statement

**Kousuke Watanabe:** Conceptualization, Data curation, Formal analysis, Investigation, Writing – original draft, Writing – review & editing. **Shinji Kohsaka:** Funding acquisition, Investigation, Methodology, Resources, Software, Writing – review & editing. **Kenji Tatsuno:** Investigation, Methodology, Resources, Software, Writing – review & editing. **Aya Shinozaki-Ushiku:** Investigation, Methodology, Resources, Writing – review & editing. **Hideaki Isago:** Conceptualization, Investigation, Writing – review & editing. **Hidenori Kage:** Investigation, Methodology, Resources, Writing – review & editing, Funding acquisition. **Tetsuo Ushiku:** Investigation, Methodology, Resources, Writing – review & editing. **Hiroyuki Aburatani:** Funding acquisition, Investigation, Methodology, Resources, Software, Writing – review & editing. **Hiroyuki Mano:** Funding acquisition, Investigation, Methodology, Resources, Software, Writing – review & editing. **Katsutoshi Oda:** Funding acquisition, Investigation, Methodology, Resources, Supervision, Writing – review & editing.

## Declaration of competing interest

The authors declare the following financial interests/personal relationships which may be considered as potential competing interests: Shinji Kohsaka reports financial support was provided by Konica Minolta Inc. Hidenori Kage reports financial support was provided by Konica Minolta Inc. Hiroyuki Aburatani reports financial support was provided by Konica Minolta Inc. Hiroyuki Aburatani reports financial support was provided by Chugai Pharmaceutical Co Ltd. Hiroyuki Mano reports financial support was provided by Konica Minolta Realm. Katsutoshi Oda reports financial support was provided by Konica Minolta Inc. Katsutoshi Oda reports financial support was provided by Chugai Pharmaceutical Co Ltd. Hiroyuki Mano reports a relationship with Daiichi Sankyo Inc that includes: funding grants. Hiroyuki Mano reports a relationship with PFDeNA that includes: funding grants. Hiroyuki Mano reports a relationship with Ambry Genetics Corp that includes: funding grants. Hiroyuki Mano reports a relationship with Ono Pharmaceutical Co Ltd that includes: funding grants. Hiroyuki Mano reports a relationship with CureGene that includes: board membership. Katsutoshi Oda reports a relationship with AstraZeneca Pharmaceuticals LP that includes: funding grants and speaking and lecture fees. Katsutoshi Oda reports a relationship with Takeda Pharmaceutical Company Limited that includes: funding grants and speaking and lecture fees. Shinji Kohsaka has patent pending to The University of Tokyo. Hiroyuki Mano has patent pending to The University of Tokyo. If there are other authors, they declare that they have no known competing financial interests or personal relationships that could have appeared to influence the work reported in this paper.

## Figures and Tables

**figs1:**
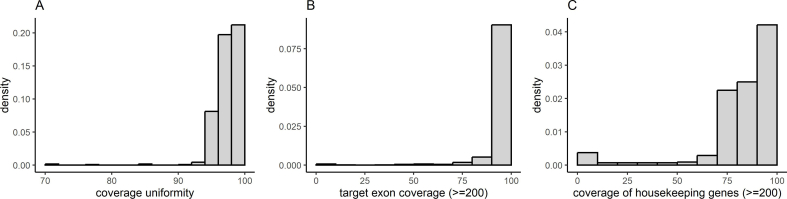


**figs2:**
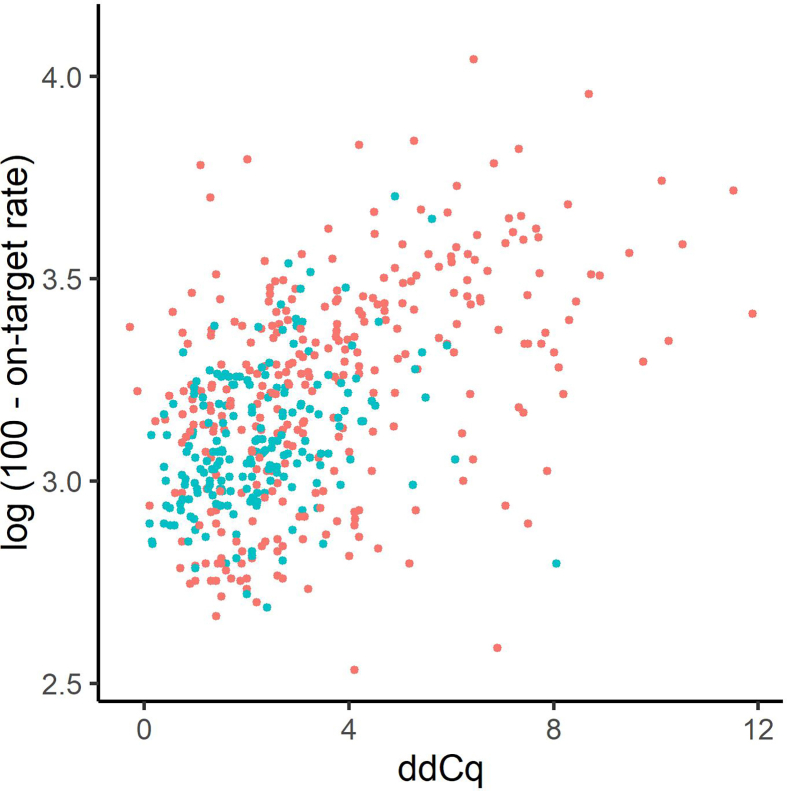


**figs3:**
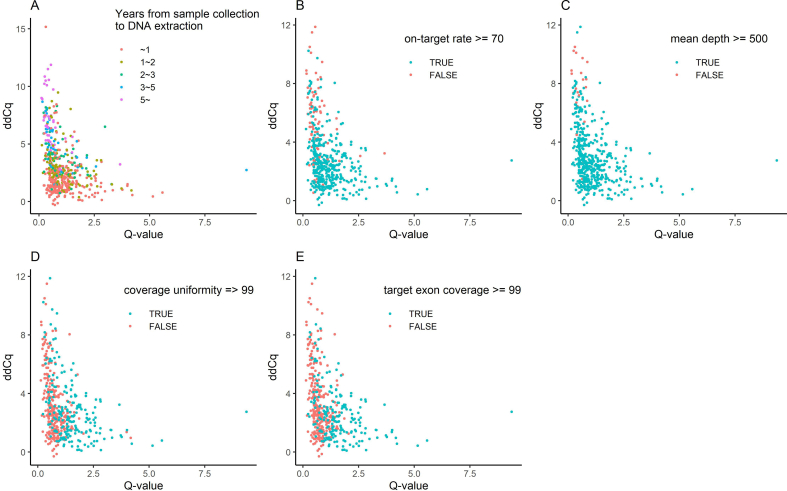


## Data Availability

The data that has been used is confidential.
