# Supplementary material for: Analysis of quality metrics in comprehensive cancer genomic profiling using a dual DNA–RNA panel
Source: Pract Lab Med. 2024 Feb 15;39:e00368. doi: 10.1016/j.plabm.2024.e00368 (PMC10883814; doi:10.1016/j.plabm.2024.e00368)
Supplement: Multimedia component 1 [file mmc1.docx]

Supplementary Figure 1 Distributions of sequencing quality metrics

Histograms depict probability densities of coverage uniformity (A), target exon coverage (B), and coverage of the housekeeping genes (C).

Supplementary Figure 2 Scatterplot illustrating the generalized linear model of on-target rate

The horizontal axis represents ddCq and the vertical axis represents log (100 – on-target rate) for samples using ideal (200 ng) input DNA. Colors indicate Q-value (blue for Q-value ≥ 1, red for Q-value < 1). This plot visually demonstrates the relationship described by the generalized linear model.

Supplementary Figure 3 Scatterplots of Q-value and ddCq

Scatterplots illustrates Q-value and ddCq of all samples (A) and samples using ideal (200 ng) input DNA (B-E). The colors of the dots represent years from sample collection to DNA extraction (A) or sequencing quality metrics (B-E).
